# Glucose Transporter 3 Is Essential for the Survival of Breast Cancer Cells in the Brain

**DOI:** 10.3390/cells8121568

**Published:** 2019-12-04

**Authors:** Min-Hsun Kuo, Wen-Wei Chang, Bi-Wen Yeh, Yeh-Shiu Chu, Yueh-Chun Lee, Hsueh-Te Lee

**Affiliations:** 1 Taiwan International Graduate Program in Molecular Medicine, National Yang-Ming University and Academia Sinica, Taipei 11529, Taiwan; rickuo626@gmail.com; 2 Institute of Anatomy & Cell Biology, National Yang-Ming University, Taipei 11202, Taiwan; 3 School of Biomedical Sciences, Chung Shan Medical University, Taichung 40201, Taiwan; changww@csmu.edu.tw; 4 Department of Urology, School of Medicine, College of Medicine, Kaohsiung Medical University, Kaohsiung 80708, Taiwan; bewen90@yahoo.com.tw; 5 Department of Urology, Kaohsiung Medical University Hospital, Kaohsiung Medical University, Kaohsiung 80708, Taiwan; 6 Brain Research Center, National Yang-Ming University, Taipei 11202, Taiwan; y27632@gmail.com; 7 Department of Radiation Oncology, Chung Shan Medical University Hospital, Taichung 40201, Taiwan; lee.yuehchun@gmail.com; 8 Taiwan International Graduate Program in Interdisciplinary Neuroscience, National Yang-Ming University and Academia Sinica, Taipei 11529, Taiwan

**Keywords:** GLUT3, breast cancer brain metastases, CREB

## Abstract

Breast cancer brain metastasis commonly occurs in one-fourth of breast cancer patients and is associated with poor prognosis. Abnormal glucose metabolism is found to promote cancer metastasis. Moreover, the tumor microenvironment is crucial and plays an active role in the metabolic adaptations and survival of cancer cells. Glucose transporters are overexpressed in cancer cells to increase glucose uptake. The glucose transporter 3 (GLUT3) is a high-affinity glucose transporter that is highly expressed in mammalian neurons. GLUT3 is also overexpressed in several malignant brain tumors. However, the role of GLUT3 in breast cancer brain metastasis remains unknown. The results of the present study demonstrated that GLUT3 is highly overexpressed in brain metastatic breast cancers and mediates glucose metabolic reprogramming. Furthermore, knockdown of cAMP-response element binding protein (CREB) could directly regulate GLUT3 expression in brain metastatic breast cancer cells. Notably, we verified and provided a novel role of GLUT3 in mediating glucose metabolism and assisting breast cancer cells to survive in the brain to promote brain metastasis.

## 1. Introduction

The tumor microenvironment is important for cancer cell survival and tumor progression [1]. As different microenvironments have various effects on tumor metastasis, cancer cells tend to have different patterns of gene expression to adapt to differing conditions when metastasis occurs [2,3,4]. Phenotypic plasticity could manifest after the occurrence of metastasis and could indicate the development of cancer cells at proximal or distal metastatic sites [5,6]. However, it remains unclear how the microenvironment can trigger tumor cell metastasis to specific organs.

Cellular energy metabolism is a potent factor in the tumor microenvironment [7]. Moreover, aberrant energy metabolism has been recognized as a hallmark of cancer [8]. Several studies have demonstrated that glucose transporters are upregulated when glucose metabolism is dysregulated. However, only a limited number of studies evaluated the effects of specific glucose transporters on the regulation of glucose metabolism. Hence, it is crucial to understand the potential response and metabolic interactions between metastatic cancer cells and the brain microenvironment.

According to previous investigations, glucose uptake in cells is mainly facilitated by the 14 members of the functional glucose transporter (GLUT) family (glucose transporter 1 (GLUT1) to glucose transporter 14 (GLUT14)). Furthermore, glucose transporters are expressed in a tissue-specific manner: GLUT1 is expressed in all tissues, glucose transporter 2 (GLUT2) in the liver, and GLUT3 in the brain [9,10]. Overexpression of glucose transporters not only increases glucose uptake by cancer cells but also acts a hallmark of tumorigenesis. Previous studies have reported that GLUT1 is overexpressed in several types of cancer, such as breast and lung cancer. Overexpression of glucose transporters not only promotes glucose metabolism but also enhances other tumorigenesis features, like immune escape [11,12]. GLUT3 is highly expressed during brain cancer tumorigenesis due to the lack of nutrients in the brain [13,14]. Moreover, despite the effects on glucose uptake, GLUT3 upregulation enhances the ability of primary brain cancer cells to resist certain chemotherapy drugs that could inhibit cancer cell angiogenesis [15]. Extracellular stimulation can cause long-lasting effects, including the promotion of cell survival or death, by triggering serial signaling cascades that ultimately converge on nuclear transcription factors [16]. One transcription factor, cAMP-response element binding protein (CREB), has a crucial role in brain development and plays a critical role in brain cancer tumorigenesis [16,17]. Furthermore, the expression of CREB is involved in several signaling pathways during tumorigenesis, such as regulation of the phosphoinositide 3-kinase (PI3k) and mitogen-activated protein kinase (MAPK) pathways, which are thought to be critical in tumor energy metabolic reprogramming [18,19]. In glioblastomas, CREB was found to regulate the growth of cancer cells via transcriptional control of miRNA-23a and Neurofibromatosis type I (Nf-1) [20,21]. In the brain, CREB is important for neuron development and survival [22,23]. Importantly, several studies have revealed that CREB plays a crucial role in the promotion of neuronal survival and adaptation in response to environmental stress and the regulation of brain responses to calorie restriction. GLUT3, the glucose transporter that is widely used by neurons, can be transcriptionally induced by CREB [24].

Based on these important clues, firstly, we aimed to determine whether GLUT3 is important for breast cancer cell survival and adaptation in the brain microenvironment and whether GLUT3 mediates cancer metabolic reprogramming. Secondly, we aimed to investigate whether the expression of GLUT3 and CREB plays a fundamental role in the promotion of breast cancer brain metastasis.

## 2. Materials and Methods

### 2.1. Cell Lines and Cell Culture

MDA-MB-231 and BT474 cells were obtained from American Type Culture Collection. The cells were seeded at a density of 2 × 10^5^ cells/well and then cultured in Dulbecco’s modified eagle medium (Corning Inc., New York, NY, USA) supplemented with 10% fetal bovine serum and 1% penicillin/streptomycin in a 37 °C incubator with a humidified atmosphere of 5% CO_2_/95% air. MDA-MB-231BR and BT474 BR cells were isolated from the brain tissue of female athymic BALB/c nude mice that received intracardial injections of WT cells as described before [25].

### 2.2. Animals and Breast Cancer Cell Brain Metastasis Model

Pathogen-free female athymic BALB/c nude mice were purchased from the Animal Production Area of the National Cancer Institute, Frederick Cancer Research Facility (Frederick, MD, USA). The mice were kept in facilities approved by the Association for Assessment and Accreditation of Laboratory Animal Care in accordance with the current regulations and standards of the Animal Center of National Yang-Ming University. Six-week-old mice were injected with 5 × 10^5^ MDA-MB-231 and BT474 cells in the heart’s left ventricle. BR cells were collected from brain tissues as described before [25].

### 2.3. Cell Viability Assay

Cell survival was analyzed by performing an MTT assay using 3-(4,5-dimethylthiazol-2-yl)-2,5-diphenyltetrazolium bromide (MTT). Briefly, MTT was added to a final concentration of 50 μg/mL and incubated at 37 °C with a humidified atmosphere of 5% CO_2_/95% air for 4 h. After incubation, the medium was carefully aspirated to avoid the removal of formazan. Approximately 50 μL/mL of DMSO was added to each well and mixed thoroughly by pipetting. After 10 min of solubilization in the culture incubator, the sample was mixed, and absorbance was read at 540 nm using multimode microplate readers (Infinite 200, TECAN, Tecan Trading AG, Switzerland).

### 2.4. Western Blotting

A total of 50 μg of cell lysate were resolved using 10% sodium dodecyl sulfate–polyacrylamide gel electrophoresis and blotted electrophoretically to polyvinylidene fluoride membranes. The membranes were then incubated with primary antibodies, and immunoreactivity was detected using horseradish-conjugated secondary antibody and visualized using enhanced chemiluminescence. The following primary antibodies were used: Rabbit anti-GLUT1 (1:500 Santa Cruz, CA, USA), mouse and rabbit anti-GLUT3 (1:500 Santa Cruz, 1:1000 Abcam), rabbit anti-pCREB (1:1000 Abcam), rabbit anti-CREB (1:1000 Abcam), rabbit anti-N-cadherin (1:1000 Abcam), rabbit anti-E-cadherin (1:500 Santa Cruz), mouse anti-HK2 (1:500 Santa Cruz), mouse anti-Ki-67 (1:500 Santa Cruz), and mouse anti-β-actin (1:1000, Abcam). Secondary antibodies (a donkey anti-goat IgG antibody (1:10,000, Jackson lab, PA, USA), a goat anti-mouse IgG antibody (1:10,000, Jackson lab), and a goat anti-rabbit IgG antibody (1:10,000, Jackson lab)) were coupled to horseradish peroxidase (HRP) for 1 h at room temperature. 

### 2.5. Immunochemistry

The human tissue array and mouse brain slides were evaluated by immunochemistry through Dako kits (Dako EnVisionTM+ Dual Link System-HRP, Agilent, CA, USA). The procedure was conducted following the manufacturer’s instruction. All antibodies were incubated with samples at 4 °C overnight followed by the HRP incubation for 1 h at room temperature. After the staining, the slides were imaged and analyzed to observe the GLUT3 expression. 

### 2.6. Production of Stably Transfected Cell Lines

The GLUT3 and CREB knockdown plasmid (clone ID: TRCN0000043616 for GLUT3 and clone ID: TRCN0000226467 and TRCN0000020343 for CREB) was purchased from RNAi Core Lab (Academia Sinica, Taiwan). In brief, before cell transfection, the plasmid and transfection reagent were gently mixed and incubated for 20 min at room temperature. The plasmid and transfection reagent mixture were added to the cells in a dropwise manner followed by incubation of cells for 72 h. The transfected cell was grown in a medium containing 2 μg/mL puromycin for selection. Twenty-one days after selection, the transfected cells were collected and analyzed for protein expression. After protein expression verification, the cells were expanded for experimental use. 

### 2.7. Glucose Uptake Ability Assay

2-(*N*-(7-nitrobenz-2-oxa-1,3-diazol-4-yl) amino)-2-deoxyglucose) (2-NBDG) was used to examine the glucose uptake. Cells were seeded at a density of 2 × 10^5^ per well in 35-mm wells and allowed to attach over 24 h. The culture medium was then replaced with a fresh medium with 2-NBDG 24 h after seeding and imaged under a fluorescent microscope with excitation and emission wavelengths of 488 and 540 nm, respectively, to determine the optical density. In the drug treatment experiment, 2-NBDG was added to the sample 1 h after ascorbic acid treatment. After 24 h, the cells were imaged under a fluorescent microscope or measured by a microplate reader with excitation and emission wavelengths of 465 and 540 nm, respectively. 

### 2.8. Lactate Detection Assay

For lactate detection, the lactate assay kit (BioVision, Inc., Milpitas, CA, USA) was used. At day 1, the cells were seeded as above, 24 h after seeding. The culture medium was replaced with DMEM medium without FBS and incubated for 24 h. The medium was renewed and centrifuged at 1000 rpm for 10 min, and the supernatant used to detect lactate dehydrogenase activity according to the manufacturer’s instructions. At first, 50-μL test samples with lactate assay buffer were prepared in a 96-well plate. The reaction mix contained 46 μL of lactate assay buffer, 2 μL of probe, and 2 μL of the enzyme mix. The solution was mixed well before use. Approximately 50 μL of the reaction mix were added to each well containing the test samples; the solution was mixed well and incubated for 30 min while protected from the light. Fluorescence (ex/em = 535/590 nm) was measured using a microplate reader. 

### 2.9. Cell Migration and Invasion Assay

Cell migration and invasion assay were conducted using transwell and transwell matrix-coated chambers with 8-µm pore-size membranes. Before the experiment, the matrix-coated transwells were stored in the incubator at a temperature of 37 °C overnight. Then, the transwells with fresh DMEM medium (without FBS) were placed in the cell incubator for 2 h. After 2 h, the cells were seeded in the transwell membrane at a density of 2.5 × 10^4^ per well and subsequently starved in culture medium without FBS both inside and outside the transwell. FBS (10%) was added to the media in the upper chamber of the transwell. The migration assay lasted for 12 h and the invasion assay lasted for 24 h. After 12 and 24 h, transwells were collected and wiped. After the transwells were dried, methanol was used to fix the cells for 20 min. Finally, after cell fixation, crystal violet was used to stain the cells overnight. After staining, the transwells were observed under a microscope and counted. 

### 2.10. Chromatin Immunoprecipitation Assay

Tumor cells (2 × 10^6^) were prepared for the chromatin immunoprecipitation (ChIP) assay with the ChIP assay kit (Cell Signaling Technology). The CREB-bound DNA of the *GLUT3* gene was immunoprecipitated with CREB antibodies. The DNA was then used for polymerase chain reaction (PCR) using primers designed for the amplification of the GLUT3 promoter. The PCR product was analyzed and quantified as described before [24]. The primers used were as follows:

(1) forward: 5′-TATTTTCTT CTCCTGCTTAGCT-3′ and
  reverse: 5′-AGTCATT TATAGT GTTTCCCTTC-3′ and
(2) forward: 5′-CCCAGGGTGGA GAGAGTGGAAG-3′ and
  reverse 5′-TTATAATCTCCGCAA AGGGTGGAG-3′ and
(3) forward: 5′-GTCATATCCC AGCGAGACCC AG-3′ and
  reverse: 5′-C GCTGTAATCTAA TTCAAGTCTTCAAG-3′.

### 2.11. Statistical Analysis

The significance of the data from patient specimens was determined by *X*^2^ test and Spearman’s rank correlation test. The significance of the in vitro data and in vivo data was determined by Student’s *t*-test (two tailed) and Mann–Whitney test (two tailed), respectively. Two-sided *p*-values less than 0.05 were considered significant. 

## 3. Results

### 3.1. Overexpression of GLUT3 in Breast Cancer Patients with Brain Metastasis

**Figure 1:**
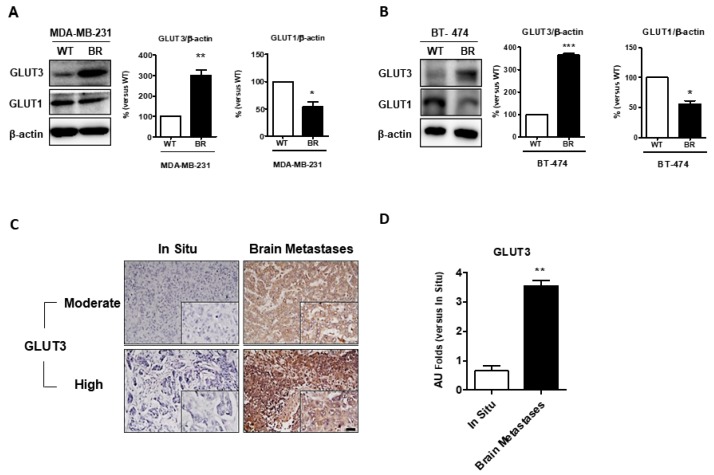
Expressions of glucose transporter 3 in breast cancer cells and commercial human tissue array in vitro; (**A**,**B**) Protein expression of GLUT3 and GLUT1 were detected by Western blot analysis in WT and BR of MDA-MB-231 and BT474 cells. The right panels show the quantification of protein expression levels. (**C**) Immunohistochemical staining for GLUT3 performed in commercial human tissue array slides. (**D**) The statistical result for tissue array data. Data are expressed as mean ± SEM from 3 to 5 independent experiments and 10 different patients sample dots in human results. * *p* < 0.05; ** *p* < 0.01; *** *p* = 0.001; scale bar = 100 μm.

At first, we investigated whether glucose transport and utilization are altered in breast cancer brain metastases. We analyzed the GLUT protein expression in parental cells (WT) and brain metastatic breast cancer cells (BR). The Western blotting analysis showed that GLUT3 is overexpressed in MDA-MB-231 and BT474 BR cells (Figure 1A,B, *p* < 0.01 and *p* < 0.001). Interestingly, in contrast to GLUT3, the expression of GLUT1 was comparably decreased in brain metastatic breast cancer cells (Figure 1A,B, *p* < 0.05). Next, we used the commercial human breast cancer metastasis tissue array (US Biomax Inc., Derwood, MD, USA, BR10011, GL861) to examine the GLUT3 expression between breast cancer patients with brain metastasis and those with primary breast cancer in situ. The immunochemistry stain indicated that the breast cancer patients with brain metastasis had significantly higher levels of GLUT3 expression than those with primary breast cancer (Figure 1C,D, *p* < 0.01). These data suggest that the expression level of GLUT3 was upregulated in breast cancer patients with brain metastasis.

### 3.2. Metabolic Reprogramming Is Upregulated in Brain Metastatic Cancer Cells

**Figure 2:**
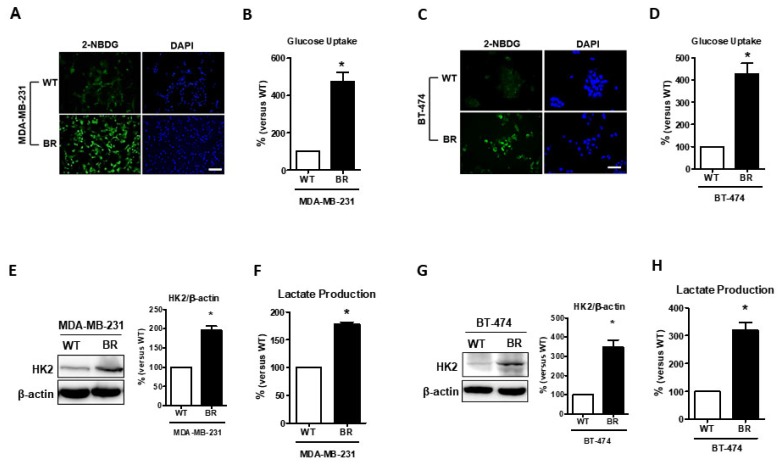
Glucose metabolic reprogramming examination in brain metastatic cells. (**A**,**C**), MDA-MB-231 and BT474 cells were treated with 2-NBDG and imaged. (**B**,**D**), 2-NBDG staining was statistically analyzed. (**E**,**G**), Western blotting showing the expression of hexokinase 2 in MDA-MB-231 and BT474 cells, and histograms representing quantification of western blotting data. (**F**,**H**), histogram showing quantification of lactate production that was detected by fluorescence microscopy. Data are expressed as mean ± SEM from three to five independent experiments. * *p* < 0.05; scale bar = 50 μm.

Recently, abnormal metabolism was identified as a hallmark of cancer progression [26]. Next, we investigated the differences in metabolic alternations between MDA-MB-231BR and wild-type cells. 2-NBDG, a glucose analog with fluorescence, was examined to determine the glucose uptake ability in vitro. Results showed that both MDA-MB-231BR and BT474BR cells had a higher affinity for glucose uptake than their WT counterparts (Figure 2A–D, *p* < 0.05). Furthermore, we determined whether the glucose utility in BR cells was more intensive. Hexokinase 2 (HK2) is considered a key mediator of aerobic glycolysis and is a hallmark of brain metastases [27,28]. Our data (Figure 2E,G, *p* < 0.05) showed that the level of HK2 expression was significantly elevated in both MDA-MB-231BR and BT474 BR cells compared with their WT counterparts. In addition, we examined whether aerobic glycolysis can result in the production of lactic acid. Our data showed that lactate production was also elevated in both MDA-MB-231BR and BT474 BR cells (Figure 2F,H, *p* < 0.05). Taken together, our findings showed that glucose metabolism is indeed more intensive in breast cancer brain metastatic cells.

### 3.3. GLUT3 Knockdown Decreased Metabolic Reprogramming

**Figure 3:**
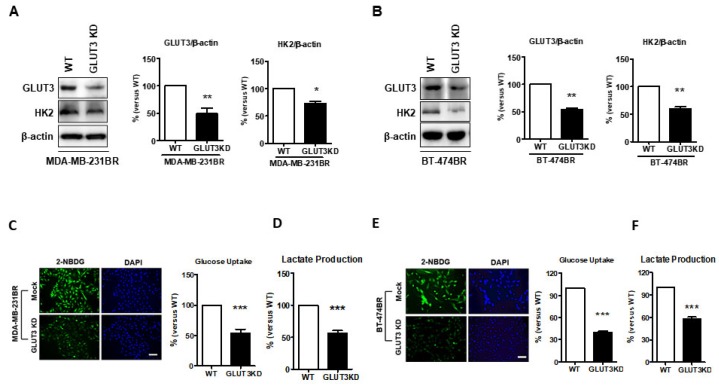
Altered glucose metabolism in GLUT3 knockdown MDA-MB-231 and BT474 brain metastatic cells; (**A**,**B**) Western blots showing the GLUT3 knockdown and reduced HK2 expression. The histograms show quantification of respective western blots. (**C**,**E**) Glucose uptake was assessed by 2-NBDG treatment that was imaged and quantified as in Figure 2B,D. (**D**,**F**) Histograms show quantification of lactate production that was detected by fluorescence microscopy. Data are expressed as mean ± SEM from three to five independent experiments. * *p* < 0.05; ** *p* < 0.01; *** *p* < 0.001; scale bar = 50 μm.

Our previous results showed that GLUT3 was upregulated in breast cancer brain metastatic cells, but whether GLUT3 could influence glucose metabolism in breast cancer brain metastases was evaluated next. The knockdown of GLUT3 with shRNA was carried out in both MDA-MB-231 BR and BT474BR cells. We found that the HK2 expression was significantly decreased after GLUT3 knockdown (Figure 3A,B, *p* < 0.05 and *p* < 0.01). Furthermore, following the GLUT3 knockdown and HK2 reduction, glucose uptake and lactate production were examined. The results demonstrated that glucose uptake and lactate production were decreased in both MDA-MB-231 BR GLUT3-knockdown cells and BT474 BR GLUT3-knockdown cells (Figure 3C–F, *p* < 0.001). Our data suggest that GLUT3 knockdown could impact glucose metabolism in brain metastatic breast cancer cells. 

### 3.4. GLUT3 Promotes Brain Metastatic Cancer Cell Malignancy

**Figure 4:**
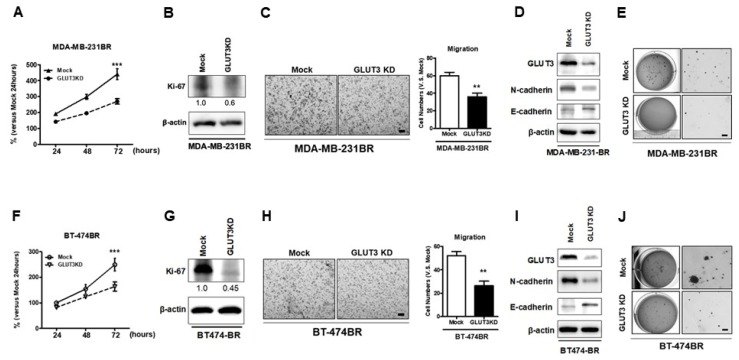
Examination of malignant cells in both GLUT3 knockdown MDA-MB-231 and BT474 brain metastatic cells; cells’ proliferative ability was examined by the MTT assay (**A**,**F**) and Ki-67 expression analyzed by western blots (**B**,**G**). The cell migration assay was performed using an 8-μm transwell chamber (**C**,**H**). The histograms show the number of cells that were counted in (**C**,**H**). Expression of N-cadherin and E-cadherin in mock and GLUT3 knockdown cells (**D**,**I**). The cells’ colony-forming ability was evaluated by soft agar colony formation assay and imaged (**E**,**J**). Data are expressed as mean ± SEM from three to five independent experiments. ** *p* < 0.01; *** *p* < 0.001; scale bars: 100 μm in migration images and 200 μm in scanned images of colony formation assays.

Next, we wanted to verify whether the GLUT3 knockdown that impacted glucose metabolism would affect cells’ malignant behaviors. The GLUT3 knockdown groups were assessed for cell malignancy. To investigate the proliferation rate of GLUT3-knockdown cells, the MTT assay and immunoblotting of Ki-67 were performed. The results showed that both MDA-MB-231BR and BT474BR GLUT3 knockdown cells were less proliferative than the mock groups (Figure 4A,B,F,G, *p* < 0.001). The cell mobility and colony-forming ability of respective GLUT3 knockdown cells were also examined. To examine cell mobility, the transwell migration assay was utilized. Both the MDA-MB-231BR and BT474BR GLUT3 knockdown groups showed a reduction in mobility compared with the mock groups (Figure 4C,H, *p* < 0.01). In addition, we examined the levels of N-cadherin and E-cadherin in cells with GLUT3 knockdown. Similar to the transwell experiments, the levels of N-cadherin, the mobility marker, were decreased after GLUT3 knockdown. Conversely, the adhesive marker, E-cadherin, levels were elevated (Figure 4D,I). Moreover, the GLUT3 knockdown groups formed less colonies than the mock groups (Figure 4E,J). These results demonstrated that knockdown of GLUT3 could interfere with the biological processes of BR of migration and malignant transformation. 

### 3.5. Survival Curves of Nude Mice That Were Intracranially Injected with GLUT3 Knockdown Brain Metastatic Cells

**Figure 5:**
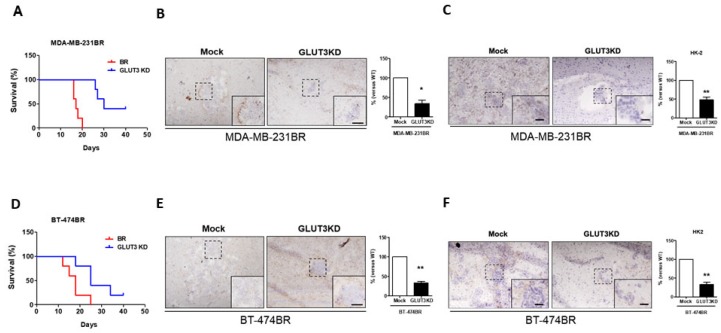
Survival curves of mice that were intracranially injected with BR and GLUT3 knockdown cells; (**A**,**D**) The survival curves indicated the life span of mice injected with MDA-MB-231 and BT474 GLUT3 knockdown and mock cells. (**B**,**E**) Brain smears were immunostained for GLUT3, photographed, and quantified for GLUT3 expression. (**C**,**F**) HK2 expression and quantification in mice brains injected with mock and GLUT3 knockdown cells. Data are expressed as mean ± SEM from five independent experiments and mice. * *p* < 0.05; ** *p* < 0.01; scale bar = 50 μm.

After verifying the glucose metabolic signaling and cell migration and malignant transformation in vitro, we investigated whether the GLUT3 knockdown could inhibit breast cancer cell survival and adaption in the mouse brain. Therefore, GLUT3 knockdown stable cells were intracranially injected into the mouse brain. Our data indicated that mice that were injected with MDA-MB-231BR and BT474BR GLUT3 knockdown cells showed a two-fold longer survival than mice that were injected with mock BR cells (Figure 5A,D). Immunochemical staining showed that the cancer volume and GLUT3 signals in mice injected with MDA-MB-231BR and BT474BR GLUT3 knockdown cells were smaller and lesser than the signals from the corresponding mock groups (Figure 5B,E, *p* < 0.01 and *p* < 0.005). Furthermore, immunochemical staining of HK2 was carried out to observe whether the metabolic signaling was alternated in the mice’s brains. Similar to the in vitro results, the tumor size and HK2 expressions were reduced in GLUT3 knockdown cells (Figure 5C,F, *p* < 0.01). Therefore, these results demonstrated and confirmed that GLUT3 is crucial for the survival of breast cancer cells in the brain. 

### 3.6. GLUT3 Overexpression Promotes Metastatic Signaling in Brain Metastatic Cells

**Figure 6:**
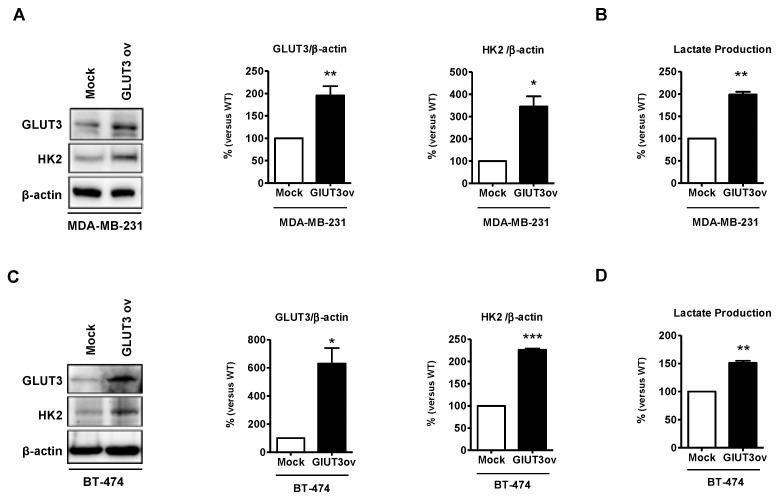
Metabolic signaling elevated in GLUT3-overexpressed parental breast cancer cells; (**A**,**C**) Western blotting demonstrating overexpression of GLUT3 and HK2 elevation. Histograms show quantification of the respective protein. (**B**,**D**) Quantification of lactate production of mock and GLUT3-overexpressed cells detected using fluorescence microscopy. Data are expressed as mean ± SEM from three to five independent experiments and mice. * *p* < 0.05; ** *p* < 0.01; *** *p* < 0.001.

GLUT3 upregulation promotes metabolic reprogramming and GLUT3 elevation in GBM-initiating tumor cells [14]. To further determine whether GLUT3 plays a role in glucose metabolic signaling in breast cancer cells, the parental MDA-MB-231 and BT474 cells were transfected with GLUT3 overexpression plasmids to establish GLUT3-overexpressed stable cells. The Western blotting showed that HK2 expression was upregulated due to the overexpression of GLUT3 in both MDA-MB-231 and BT474 cells (Figure 6A,C, *p* < 0.01 and *p* < 0.001). Importantly, the GLUT3-overexpressed groups showed an increase in the production of lactate (Figure 6B,D, *p* < 0.01). In short, our finding demonstrated that overexpression of GLUT3 could mediate and enhance glucose metabolism. 

### 3.7. GLUT3 Overexpression in Parental Breast Cancer Cells Causes Breast Cancer Cells to Become Malignant

**Figure 7:**
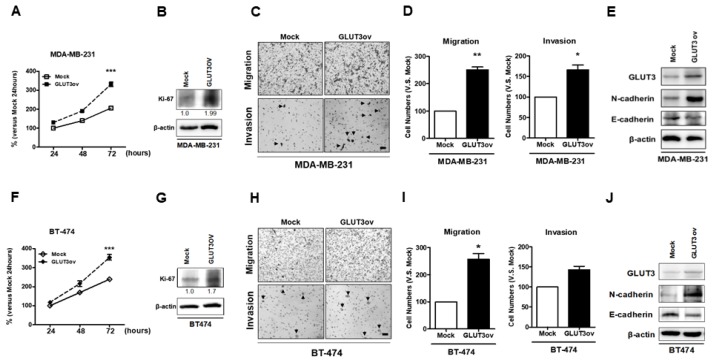
Examination of cell malignant behaviors of the parental breast cancer cells after GLUT3 overexpression; (**A**,**F**) The proliferative abilities of parental cells after GLUT3 overexpression were determined by MTT. (**B**,**G**) the Ki-67 expressions by western blots. (**C**,**H**) The photomicrographs showing the mobile and invasive abilities by transwell migration assay and collagen-coated transwell assay (the arrowhead indicates invasive cells), (**D**,**I**) Histograms showing quantification of data in (**C**,**H**). (**E**,**J**) The expression of N- and E-cadherin in mock and GLUT3-overexpressed cells. The data are expressed as mean ± SEM from five independent experiments. * *p* < 0.05; ** *p* < 0.01; *** *p* < 0.001; scale bar = 100 μm.

After confirming that GLUT3 overexpression could mediate metabolic reprogramming in parental breast cancer cells, we evaluated whether GLUT3-overexpressed cells exhibit more malignant behavior. Firstly, the MDA-MB-231 and BT474 GLUT3-overexpressed cells showed higher proliferative ability than the mock cells as indicated by the MTT assay and immunoblotting of Ki-67 (Figure 7A,B,F,G, *p* < 0.001). Furthermore, the cell mobility and invasion were determined in stable GLUT3-overexpressed cells. Our data showed that GLUT3 overexpression resulted in higher mobility and invasive ability compared to the mock groups (Figure 7C,D,H,I, *p* < 0.05 and *p* < 0.01). In addition, GLUT3-overexpressed cells showed a higher level of mobility markers, N-cadherin (Figure 7E,J). Therefore, these findings suggest that overexpression of GLUT3 can significantly enhance the malignant behavior of cancer cells.

### 3.8. GLUT3 Expression Is Mediated by CREB in Brain Metastatic Cells

**Figure 8:**
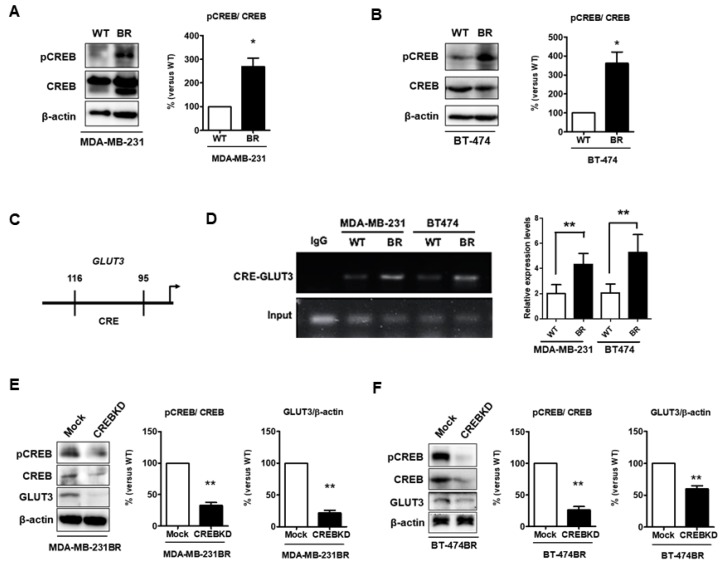
Levels of pCREB are increased and CREB mediates GLUT3 expression in breast cancer brain metastatic cells; (**A**,**B**) pCREB and CREB expression determined by Western blotting in both MDA-MB-231 and BT474 cells, and histograms showing quantification of respective western blots. (**C**) The schematic CREB binding region in GLUT3 promoter. (**D**) The CREB binding with GLUT3 promoter DNA was assessed by RT-PCR. The histogram shows quantification of RT-PCR data. (**E**,**F**) the CREB knockdown validation and GLUT3 reduction were analyzed by Western blotting. The histograms show quantification of each protein in respective western blots. Data are expressed as mean ± SEM from three to five independent experiments and mice. * *p* < 0.05; ** *p* < 0.01.

CREB can be a potent regulator of neural progenitor cell survival and expansion in the tumor microenvironment [16]. Furthermore, CREB regulates the expression of GLUT3 in neurons [24]. We speculated that CREB was involved in regulating GLUT3 expression to promote breast cancer survival in the brain microenvironment. Initially, we found that phospho-CREB, the active form of CREB, was significantly higher in both MDA-MB-231 and BT474 brain metastatic breast cancer cells (Figure 8A,B, *p* < 0.05). Based on our previous results (Figure 1A,B), CREB and GLUT3 were both elevated in BR cells. The ChIP assay was used to assess whether CREB binds to GLUT3 promoter [24] to modulate GLUT3 expression in breast cancer cells to promote brain metastasis. The promoter region is illustrated in Figure 8C, and the data showed that the level of CREB expression significantly increased after binding with the CRE region (−116 to −95 bp) of GLUT3 promoter (Figure 8D). Next, we examined the role of CREB in the regulation of GLUT3. A CREB knockdown assay was performed in BR cells. The results showed that the level of GLUT3 expression was significantly decreased in the CREB knockdown BR groups (Figure 8E,F, *p* < 0.01 and *p* < 0.001). In summary, these results illustrated that CREB is involved in the direct regulation of GLUT3 expression in breast cancer brain metastatic cells.

## 4. Discussion

To investigate the role of glucose transporters in metastatic cells, we referred to the results of a previous study [25] investigating the molecular regulation of cancer cells at the metastasis site [25]. Several prior investigations indicated that some glucose transporters would be overexpressed during tumorigenesis. In our study, we not only discovered the overexpression of the specific glucose transporter, GLUT3, but we also found that dominant glucose transporters would be changed in breast cancer after brain metastasis. In Figure 1, we found the importance of GLUT3, which is seldom expressed in the breast [29], but it was overexpressed in MDA-MB-231 BR cells after brain metastasis. GLUT1 expression was decreased in BR groups. Based on the results of a previous study, the expression of some cancer genes is altered after metastasis [4]. Therefore, the alternations in this study might involve some genetic or epigenetic modifications after breast cancer brain metastases; however, additional studies are warranted to confirm this finding. 

GLUT3 has the highest glucose affinity among all class I glucose transporters and is the most well-identified class in the glucose transporter family [9,10]. As noted above, GLUT3 is seldom expressed in the breast and less overexpressed in breast cancer patients than GLUT1 [29,30]. However, GLUT3 is found to be highly expressed in certain cancer types. GLUT3 expression is found to be high in non-small cell lung cancer and that correlates to poor prognosis compared to other lung cancer types [31]. In addition, *SLC2A3*, the gene of GLUT3, is upregulated in high mortality colorectal carcinoma patients based on the TCGA dataset [32]. Additionally, in the brain, GLUT3 is also overexpressed in aggressive brain cancer cells and could impact the drug resistant ability [14,15]. Based on these findings, it appears that GLUT3 is potentially required in high glucose-demanding malignant cells. Besides, Chang et al. demonstrated that tumors can enhance their own glucose uptake, making them a highly competitive energy source in the metastatic microenvironment [33]. Moreover, using a glucose transporter with a higher glucose affinity could help breast cancer cells survive in the brain. Cancer cells have the ability of increasing their glucose metabolism, which can induce proliferation and other processes to maintain their survival. Furthermore, the consumption of glucose by cancer cells could be observed [8,26]. Metastatic cells expressed high-affinity glucose transporters; however, it remains unclear whether high-affinity glucose transporters could enhance the glucose uptake ability of breast cancer brain metastatic cells. The 2-NBDG assay results demonstrated that metastatic cells have higher glucose uptake. We also verified that oncogenic glucose metabolic enzyme HK2 expression and the aerobic glycolysis final product, lactate, were both significantly higher in BR cells. As aerobic glycolysis is recognized as a hallmark property of malignant cells, our results indicate that brain metastatic cells have a greater ability to reprogram their metabolism in order to survive in the brain. Although several studies demonstrated that glucose consumption would be elevated during tumorigenesis, very few studies indicated that glucose uptake is increased in metastatic cells. Here, we demonstrated that glucose uptake and metabolism were significantly increased in brain metastatic cells. 

The glucose reprogramming phenomenon is common in patients with cancer, and targeting the glucose metabolism pathway in cancer therapies has been demonstrated in multiple previous studies [7]. We found that overexpression of GLUT3 in BR cells results in a higher ability to metabolize glucose. We validated GLUT3’s role in mediating BR cell survival, and found glucose metabolic ability was lower in GLUT3 knockdown BR cells. Moreover, the results of the cell behavior analysis indicated that GLUT3 knockdown groups were less malignant, with lower migration and invasion. Furthermore, the mice injected with GLUT3 knockdown cells in their brain had a longer life span. Previous studies showed that the production of lactate is increased during cancer progression, and this could lead to a reduction in the immune response and consequently increase the survival of cancer cells [34,35]. In summary, our results indicate that GLUT3 loss could ameliorate not only cancer metabolic reprogramming but also brain metastatic breast cancers. Interestingly, and as expected, GLUT3 overexpression influenced the expression of HK2. Moreover, GLUT3-overexpressed cells were more malignant after GLUT3 expression increased. This is the first study to report that overexpressed GLUT3 could initiate metabolic changes in breast cancer cells. As Mark Masin et al. illustrated in their study on non-small cell lung cancer, GLUT3 is induced during epithelial–mesenchymal transition (EMT). In this study, the results demonstrated that EMT is not only induced, but the lung cancer cells that overexpressed GLUT3 exhibited higher glucose import [31]. Although Mark Masin et al. did not emphasize the possible occurrence of brain metastasis, 40% of non-small cell lung cancer patients develop brain metastasis. It is unclear whether elevated GLUT3 expression might be a diagnostic marker of lung cancer metastasis in the brain. 

CREB plays an important role in the survival and metabolism of cells in the brain microenvironment [17]. Moreover, we found that pCREB, the functional unit of CREB, is elevated in brain metastatic cancer cells. Furthermore, GLUT3 expression was significantly decreased after CREB was knocked down in both MDA-MB-231 and BT474 cells. The ChIP assay indicated that CREB directly regulated GLUT3 expression, and CREB binding ability was higher in brain metastatic breast cancer cells. Thus, we presume that a more activated CREB would bind to GLUT3 promoter in brain metastatic cells and subsequently induce more GLUT3 expression once breast cancer cells metastasize to the brain. 

## 5. Conclusions

In conclusion, we demonstrated GLUT3 regulation of breast cancer cells survival and metastasis to the brain. Therefore, our study further illustrated that: (1) After metastasis, the expression and distribution of dominant glucose transporters is altered so that cancer cells can survive in the brain, (2) GLUT3 plays a potent role in breast cancer brain metastasis, (3) GLUT3 expression is regulated by CREB, and (4) GLUT3 might be a potential target for the treatment of breast cancer brain metastasis.
